# Deep phenotyping of dementia in a multi-ethnic cardiovascular cohort: The Multi-Ethnic Study of Atherosclerosis (MESA)

**DOI:** 10.1371/journal.pone.0298952

**Published:** 2024-04-18

**Authors:** Mohammad R. Ostovaneh, Timothy M. Hughes, Colin O. Wu, Robyn L. McClelland, Ramon Casanova, David A. Bluemke, Russell P. Tracy, Steven Shea, Susan R. Heckbert, João A. C. Lima, Bharath Ambale-Venkatesh

**Affiliations:** 1 Division of Cardiology, Department of Medicine, Johns Hopkins University, Baltimore, Maryland, United States of America; 2 Department of Gerontology and Geriatric Medicine, Wake Forest School of Medicine, Winston-Salem, North Carolina, United States of America; 3 National Heart, Lung and Blood Institute, National Institutes of Health, Bethesda, Maryland, United States of America; 4 School of Public Health, University of Washington, Seattle, Washington, United States of America; 5 Department of Radiology, University of Wisconsin, Madison, Wisconsin, United States of America; 6 Department of Pathology and Laboratory Medicine, Laboratory for Clinical Biochemistry Research, University of Vermont, Colchester, Vermont, United States of America; 7 Division of General Medicine, Department of Medicine, Columbia University, New York, New York, United States of America; 8 Department of Radiology, Johns Hopkins University, Baltimore, Maryland, United States of America; Radiation Effects Research Foundation, JAPAN

## Abstract

**Background:**

Our understanding of the specific aspects of vascular contributions to dementia remains unclear.

**Objectives:**

We aim to identify the correlates of incident dementia in a multi-ethnic cardiovascular cohort.

**Methods:**

A total of 6806 participants with follow-up data for incident dementia were included. Probable dementia diagnoses were identified using hospitalization discharge diagnoses according to the International Classification of Diseases Codes (ICD). We used Random Forest analyses to identify the correlates of incident dementia and cognitive function from among 198 variables collected at the baseline MESA exam entailing demographic risk factors, medical history, anthropometry, lab biomarkers, electrocardiograms, cardiovascular magnetic resonance imaging, carotid ultrasonography, coronary artery calcium and liver fat content. Death and stroke were considered competing events.

**Results:**

Over 14 years of follow-up, 326 dementia events were identified. Beyond age, the top correlates of dementia included coronary artery calcification, high sensitivity troponin, common carotid artery intima to media thickness, NT-proBNP, physical activity, pulse pressure, tumor necrosis factor-α, history of cancer, and liver to spleen attenuation ratio from computed tomography. Correlates of cognitive function included income and physical activity, body size, serum glucose, glomerular filtration rate, measures of carotid artery stiffness, alcohol use, and inflammation indexed as IL-2 and TNF soluble receptors and plasmin-antiplasmin complex.

**Conclusion:**

In a deeply phenotyped cardiovascular cohort we identified the key correlates of dementia beyond age as subclinical atherosclerosis and myocyte damage, vascular function, inflammation, physical activity, hepatic steatosis, and history of cancer.

## Introduction

With increases in life expectancy, due in large part to reduction of cardiovascular mortality, the burden of dementia and cognitive dysfunction to health care systems and populations has increased exponentially [1]. In 2017, dementia affected more than 47 million people globally [2] and this number is expected to reach 115 million by 2050 [3]. The costs associated with dementia care are expected to soon surpass care costs for cardiovascular diseases and cancer [4]. Although dementia research has mainly focused on Alzheimer’s disease (AD) as the most common clinical subtype, vascular disease contributions to cognitive impairment and dementia (VCID) are the second most common form of clinical dementia accounting for at least 20% of cases [1, 3]. VCID contributes to “mixed dementia”, with cerebrovascular disorders often coexisting with AD [1, 3, 5]. Moreover, in patients with milder forms of VCID, the rate of conversion to dementia and mortality is significantly increased, highlighting mild VCID as an important target for prevention of progressive cognitive decline [3, 6]. Accordingly, “prevention of vascular dementia” was identified as one of the six scientific areas of focus for the next decade in the National Heart, Lung and Blood Institutes (NHLBI) strategic vision [1, 7]. Despite the growing burden of dementia, more recent epidemiologic data suggest a decline in its incidence rate in developed countries, partly attributed to improvement in prevention and treatment of cardiovascular diseases (CVD). This raises the promise of preventing dementia through enhanced cardiovascular (CV) risk factor control [1, 8]. CV risk factors such as hypertension are strongly related to dementia and brain tissue injury [9, 10]. Our group has shown previously that left ventricular remodeling as well as NT-proBNP elevation are associated with dementia [11, 12].

Existing epidemiological cohorts provide a unique opportunity to leverage the existing data on thousands of participants with millions of phenotypic datapoints for the purpose of mining clinical data related to VCID and dementia. To this extent, statistical machine learning methods such as Random Forests have been used for data mining and biomarker discovery to identify important predictors of CVD in epidemiologic cohorts [13, 14]. In this study, we used Random Forests [15] to identify correlates of dementia and cognitive function from approximately two hundred variables from multiple distinct domains collected at the baseline examination of the Multi-Ethnic Study of Atherosclerosis (MESA).

## Methods

The detailed design of MESA has been described elsewhere [16]. Briefly, men and women 45 to 84 years old and free of overt cardiovascular disease at baseline were enrolled across 6 US field centers (2000–2002). Participants represented 4 racial/ethnic groups including 38% White, 28% Black, 22% Hispanic and 12% Chinese American. All participants provided written informed consent prior to participation and local institutional review boards approved the study protocol prior to data collection. Baseline evaluations included but were not limited to the following: Questionnaires for assessment of demographics and socioeconomic status, CVD risk factors, past medical and family history, anthropometric measurements, laboratory markers, apolipoprotein E subtypes, electrocardiogram, cardiac and aortic magnetic resonance imaging, non-contrast computed tomography for measurement of coronary artery calcium and liver fat content. In this study, we included 198 variables from these domains which had less than 40% missing values. The data underlying this article were provided by MESA coordinating center and were accessed on April 15, 2021.

### Outcomes

Participants were contacted every 9–12 months to identify incident death, interim hospitalizations, and outpatient cardiovascular disease diagnoses. Hospital discharge diagnosis codes were obtained, and medical records were reviewed by two physicians. Probable dementia, including Alzheimer disease, vascular dementia, and other dementias, was identified using hospitalization discharge diagnoses codes according to the International Classification of Diseases (ICD) Medical Diagnosis Codes, Ninth Revision: 290, 294, 331.0, 331.1, 331.2, 331.82, 331.83, 331.9, 438.0, and 780.93; ICD, Tenth Revision: F00, F01, F03, F04, G30, G31 (excluding G31.2), I69.91, and R41. Importantly, the use of hospitalization discharge ICD codes for the diagnosis of dementia has been validated and used in prior publications from MESA [11, 12, 17, 18].

Cognitive function measures included Cognitive Abilities Screening Instrument (CASI) score, as well as forward and backward Digit Span and were obtained as part of the fifth follow-up (2010–2012) MESA examination 10 to 12 years after the baseline MESA exam [11, 12].

### Statistical analysis

Data is presented as mean (SD) or frequency (%), as appropriate. Missing values were assumed to occur on random basis and were imputed within the entire dataset using the Random Forest imputation algorithm [13, 15]. The dataset was split into training (66%) and testing (33%) datasets. The training dataset was used to generate prediction models while the testing (or validation) dataset was utilized to assess model performance.

We used the random forest algorithms for analysis because of their many known advantages including robustness to correlated features, large number of input features, nonlinear relationships, and built-in cross-validation. We used RandomForestSRC package [15] in R environment to generate Competing-risk Random Survival Forest (RSF) models for efficient variable selection and identification of the most important correlates of dementia. Death and stroke were considered competing risks. RSF is an ensemble tree-based method for analysis of right-censored data [13, 15]. In this method, each tree in the forest is generated based on random bootstrapping or subsampling, a process that by default leaves out one-third of the data (out- of-bag data, OOB). The OOB samples can be used for built-in cross-validation and to generate measures of variable importance. We considered a splitting rule that was based on Gray’s test for improved prediction of cumulative event incidence [19]. An ensembled hazard function was then calculated by averaging the tree’s hazard functions. Prediction error was calculated from OOB data. We used 1000 trees and randomly selected one third of variables as candidates for splitting a node (mtry hyperparameter = 198/3) in the forest to stabilize the prediction error. Given the known strong association of age with incident dementia, we performed a sensitivity RSF analysis in participants with age greater than median (62 years old) at baseline. To construct a parsimonious model, we also conducted a competing risk Cox proportional hazard regression analysis on the top 10 correlates of dementia as identified by RSF analysis, employing a backward stepwise variable selection technique.

A similar approach using Random Forest (RF) regression was taken to identify the most important correlates of measures of cognitive function (CASI score, forward and backward digit span variables). Given significant influence of demographic and socioeconomic factors on measures of cognitive function [20], we constructed linear regression models to generate z-scores for CASI score and digit span variables adjusted for age, gender, education and race. RF analysis used the adjusted z-scores as the outcome variables. The splitting rule for RF models was based on mean squared error.

The most important features from RSF and RF models were identified using a RF measure of variable importance called permutation index (VIMP) [15, 21]. For the RSF model of incident dementia, we assessed the possibility of interaction between the top 15 variables using the VIMP method provided in the RandomForestSRC package. In this method, variables are paired and their paired VIMP is calculated, and it is compared to the additive VIMP (sum of VIMP for 2 variables). A large difference between paired and additive VIMP indicates the presence of an interaction [22].

The RSF and RF models generated using the training dataset were provided with the testing dataset as input to assess the performance of RSF [Harrell’s concordance index (C-index) and Brier score] and RF models [root mean square error(RMSE), R-squared and mean absolute error (MAE)] [23, 24]. A higher C-index and lower Brier score indicate greater performance of RSF models while lower RMSE/MAE and higher R-squared indicate higher performance for RF models.

## Results

We included 6806 MESA participants with available dementia follow-up data and randomly split the sample size to 4492 participants in the training and 2314 in the testing datasets. The mean age of participants was 62.1± 10 and 3598 (53%) were female. Baseline characteristics of participants are listed in Table 1. Of the included participants, 291 (4.3%) developed heart failure before meeting the endpoint of dementia or being censored, 630 (9.3%) had a cardiovascular event, and 805 (11.8%) developed atrial fibrillation. During 73881 person years of follow-up (median 14 years), 326 participants developed dementia, while 945 and 232 met the competing endpoints of death and stroke, respectively.

**Table 1:** Baseline characteristics of participants.

	Without incident dementia	With incident dementia
Variable	N = 6480	N = 326
Age (years), mean(SD)	61.6(10.0)	73.6(7.6)
Male, n(%)	3032(46.8)	175(53.7)
Race, n(%)		
White	2465(38.0)	154(47.2)
Black	1794(27.7)	95(29.1)
Hispanic	1436(22.2)	57(17.5)
Chinese American	784(12.1)	20(6.1)
Systolic blood pressure (mmHg), mean(SD)	126.1(21.3)	135.5(23.8)
Diastolic blood pressure (mmHg), mean(SD)	71.9(10.3)	71.5(10.0)
Pulse pressure (mmHg), mean(SD)	54.2(17.0)	63.9(19.3)
Antihypertensive use, n(%)	2368(36.6)	162(49.7)
Body mass index (kg/m^2^), mean (SD)	28.4(5.5)	27.5(4.8)
Hip circumference (cm), mean(SD)	105.7(11.5)	103.9(10.1)
Waist circumference (cm), mean(SD)	98.1(14.5)	97.9(12.8)
Weight (lbs), mean(SD)	173.7(38.4)	166.2(33.6)
Highest weight in 3 years (lbs), mean(SD)	179.3(39.9)	174.2(34.9)
Height (cm), mean(SD)	166.4(10.0)	165.3(10.4)
Total cholesterol (mg/dl), mean(SD)	194.2(35.9)	192.2(32.2)
HDL cholesterol (mg/dl), mean(SD)	50.9(14.8)	52.1(15.0)
Antihyperlipidemic use, n(%)	1037(16.0)	61(18.8)
GFR (ml/min/1.73 m^2^), mean(SD)	74.7(16.4)	69.5(16.2)
Diabetes mellitus, n(%)		
Impaired fasting glucose	884(13.7)	55(16.9)
Untreated diabetes	167(2.6)	12(3.7)
Treated diabetes	638(9.9)	36(11.1)
Serum glucose (mg/dl), mean(SD)	97.3(30.3)	99.6(28.4)
Smoking, n(%)		
Former	2354(36.5)	126(38.6)
Current	848(13.1)	39(12.0)
CAC score, mean(SD)	134.2(397.0)	365.5(655.6)
CAC volume, mean(SD)	118.3(332.6)	319.0(548.7)
hsTroponin (pg/ml), mean(SD)	6.4(7.4)	10.3(10.1)
Liver to spleen attenuation ratio, mean(SD)	1.2(0.3)	1.2(0.2)
Carotid flow velocity, mean(SD)	9.6(15.6)	9.3(18.9)
Moderate to vigorous MET/week (min/week), mean(SD)	5813.2(5947.3)	4528.7(4680.4)
Total MET, min/week (min/week), mean(SD)	3121.7(3970.1)	1000.0(2211.1)
Moderate MET/week (min/week), mean(SD)	4863.4(4498.1)	3906.5(3809.9)
Work hours/week, mean(SD)	21.7(23.9)	7.0(14.2)
Transportation MET/week (min/week), mean(SD)	845.6(831.0)	739.3(737.9)
Plasmin-antiplasmin complex (nm), mean(SD)	4.7(2.2)	5.5(2.3)
TNFsr (pg/ml), mean(SD)	1353.8(44.9)	1686.4(663.8)
IL-2sr (pg/ml), mean(SD)	968.9(447.5)	1225.8(648.4)
NT-proBNP (pg/ml), mean(SD)	95.7(193.8)	184.1(211.5)
Descending aortic strain, mean(SD)	10.9(8.2)	11.1(8.0)
Common carotid IMT (mm), mean(SD)	0.9(0.2)	1.0(0.2)
Internal carotid IMT (mm), mean(SD)	1.1(0.6)	1.4(0.7)
Carotid distensibility coefficient (1/mmHg), mean(SD)	0.01(0.01)	0.01(0.01)
Income, n(%)		
Less than $25000	1904(30.6)	151(50.0)
$25000-$75000	2891(46.5)	109(36.1)
More than $75000	1435(23.0)	42(13.9)
Education, n(%)		
Less than 12 years	2317(35.9)	142(43.6)
12 years or more	4139(64.1)	184(56.4)
Alcohol use, n(%)		
Former	1526(23.5)	94(28.8)
Current	3595(55.5)	150(46.0)
Medium HDL size (nm), mean(SD)	13.3(6.8)	12.9(6.4)
Large HDL size (nm), mean(SD)	6.0(3.4)	6.4(3.5)
Aortic distensibility, mean(SD)	1.9(1.3)	1.3(0.7)
History of cancer, n(%)	484(7.5)	51(15.7)

SD: Standard deviation; HDL: High density lipoprotein; hsTroponin: High sensitivity troponin; NT-proBNP: N-terminal-proBrain natriuretic peptide; CAC: Coronary artery calcification; TNFsr: Tumor necrosis factor-alpha soluble receptor; IL-2sr: Interleukin 2 soluble receptor; IMT: Intima-to-media thickness; GFR: Glomerular filtration rate, MET: Metabolic equivalent of task

S1 Fig in S1 Appendix shows the cumulative incidence function (CIF) and change in OOB (out of bag) prediction error by increasing the number of trees in the RSF model for incident dementia. The CIF increases over time for dementia, death and stroke consistently. The OOB prediction error decreases by increasing the number of trees, stabilizing beyond ~400 trees. The OOB prediction error for 1000 trees in the RSF model was 12% for dementia, 20% for death and 9% for stroke.

**Fig 1:**
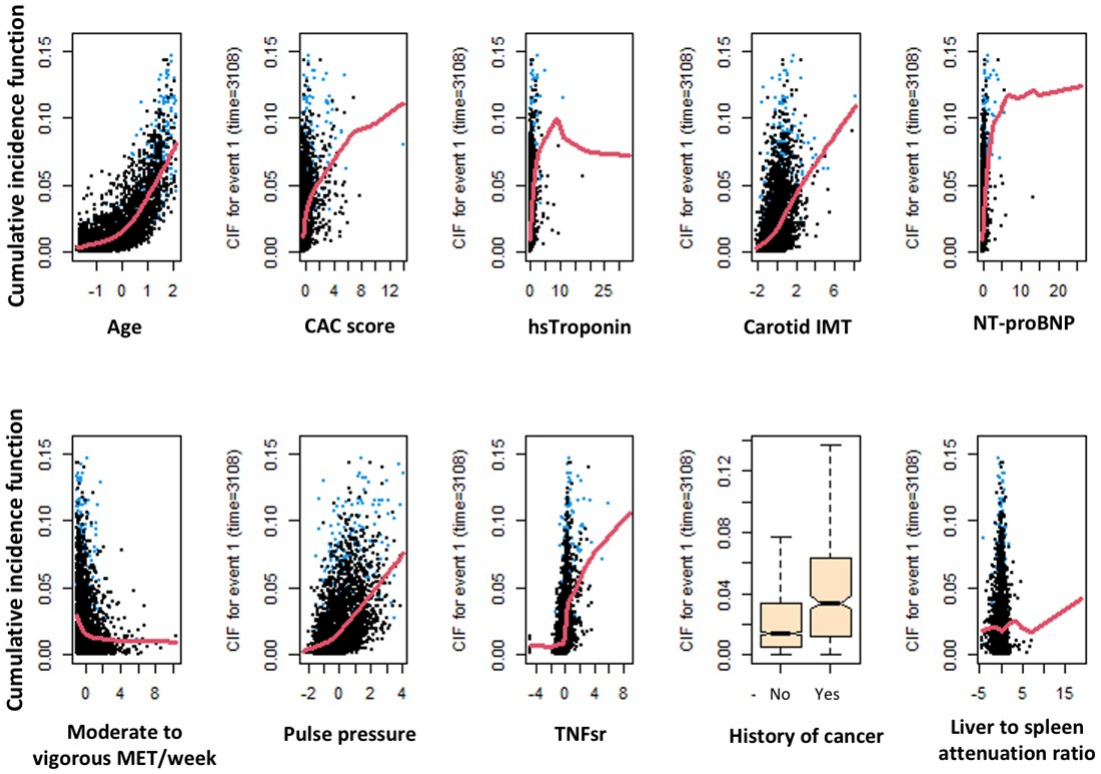
Marginal effects of top 10 variables from Random Survival Forest on cumulative incidence function of dementia. X-axes represents the standard z-score for each variable. hsTroponin: High sensitivity troponin; NT-proBNP: N-terminal-proBrain natriuretic peptide; CAC: Coronary artery calcification; TNFsr: Tumor necrosis factor-alpha soluble receptor; IL2-sr: Interleukin 2 soluble receptor; IMT: Intima-to-media thickness; MET: Metabolic equivalent of task. x-axes represent the standard values for each variable. y-axes represent cumulative incidence function for dementia.

Fig 1 shows the directionality of marginal effects for the top 10 variables on the cumulative incidence function (CIF) for dementia. No interactions were detected among the top 15 correlates of dementia. Age was by far the strongest correlate of dementia. As listed in Table 2, following age, the top correlates of dementia were higher coronary artery calcification (CAC volume), subclinical myocyte damage indexed as high sensitivity troponin (hsTroponin), higher common carotid intima to media thickness (IMT) and NT-proBNP levels, lower moderate to vigorous physical activity indexed as metabolic equivalent of task (MET), higher pulse pressure and inflammation indexed as tumor necrosis factor soluble receptor (TNFsr), history of cancer, and hepatic steatosis indexed as the liver to spleen attenuation ratio. S2 Fig in S1 Appendix shows that top 10 correlates of dementia yielded the maximum model performance by RSF-based Harrell’s C-index. In sensitivity analysis using higher number of trees (ntrees = 5000) in RSF model (S2 Table in S1 Appendix), the top correlates of dementia are largely similar to those identified in primary analysis. In parsimonious competing risk Cox proportional hazard regression analysis on the top 10 correlates of dementia as identified by RSF analysis using a backward stepwise variable selection technique, age (hazard ratio (HR) of 3.7), CAC volume (HR of 1.05), hsTroponin (HR of 1.07), common carotid IMT (HR of 1.1), and TNFsr (HR of 1.1) emerged as important correlates of dementia. In participants 62 years or older (n = 3353, Table 2), the most important features of dementia were relatively similar to those for the entire cohort. However, in this subgroup, higher interleukin-2 soluble receptor (IL-2sr) and apolipoprotein E levels, higher internal carotid IMT, and lower descending aortic strain were also associated with dementia. Top correlates of dementia in participants younger than 62 years old is presented in S2 Table in S1 Appendix. It should be noted that event rate in younger participants is small (26 incident dementia among 3452 participants), limiting the analysis power in this subgroup.

**Table 2:** Top correlates of incident dementia based on Random Survival Forest analysis.

All participants		Participants ≥ 62 years old	
Variable	VIMP	Variable	VIMP
Age	15.486	Age	8.94
CAC volume	0.321	hsTroponin	0.714
hsTroponin	0.309	IL-2sr	0.636
Common carotid IMT	0.233	CAC volume	0.395
NT-proBNP	0.197	NT-proBNP	0.374
Moderate to vigorous MET/week	0.157	Internal carotid IMT	0.222
Pulse pressure	0.125	Apo-protein E	0.215
TNFsr	0.115	Descending aortic strain	0.212
History of cancer	0.094	Common carotid IMT	0.211
Liver to spleen attenuation ratio	0.093	CAC score	0.135

hsTroponin: High sensitivity troponin; NT-proBNP: N-terminal-proBrain natriuretic peptide; CAC: Coronary artery calcification; TNFsr: Tumor necrosis factor-alpha soluble receptor; IL-2sr: Interleukin 2 soluble receptor; IMT: Intima-to-media thickness; MET: Metabolic equivalent of task; VIMP: Variable importance

**Fig 2:**
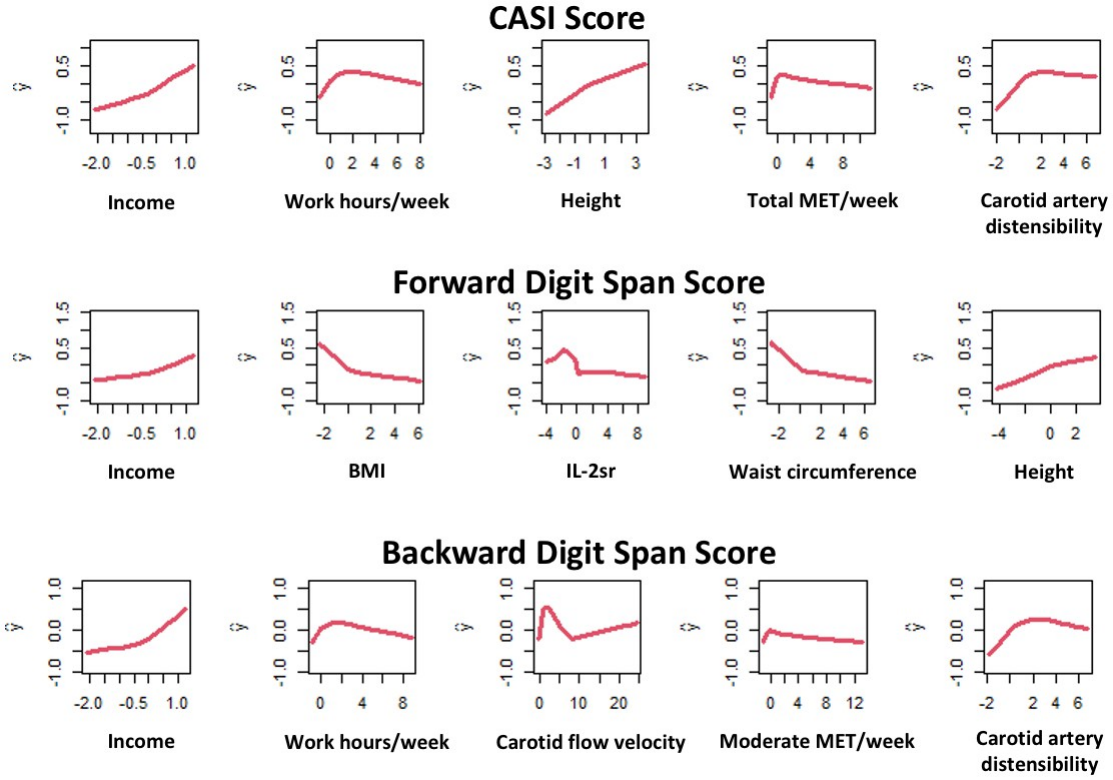
Marginal effects of top variables from Random Forest analysis on subclinical measures of cognitive function including Cognitive Abilities Screening Instrument (CASI) score, forward and backward digit span. X-axes represent the standard z-score for each variable and y-axes represent the adjusted (for age, gender, education and race) z-score for CASI score, backward and forward digit span. TNFsr: Tumor necrosis factor-alpha soluble receptor; MET: Metabolic equivalent of task; IL-2sr: Interleukin 2 soluble receptor; BMI: Body mass index; GFR: Glomerular filtration rate.

Table 3 and Fig 2 illustrate the top correlates of adjusted (for age, gender, education and race) z-scores for CASI score, backward and forward digit spans. Lower income, lower physical activity (reported working hours/week, total MET/week, reported total physical activity hours/week, transportation MET/week, moderate MET/week) and increased body size (height, body mass index, waist and hip circumference, highest weight in 3 years prior to MESA baseline examination) were highly associated with all three measures of cognitive function. Carotid flow parameters (lower carotid distensibility coefficient and carotid flow velocity) and alcohol use correlated with CASI score and backward digit span. Measures of increased inflammation (IL-2sr, TNFsr, and plasmin-antiplasmin complex) were among the important correlates of forward digit span. Furthermore, higher levels of serum glucose was associated with CASI score while reduced kidney function indexed as glomerular filtration rate was among the top correlates of backward digit span.

**Table 3:** Top variables at baseline MESA exam that are associated with measures of cognitive function at MESA exam 5.

CASI score*		Forward digit span*		Backward digit span*	
^Mean (SD): 86.9(11.4)^		^Mean (SD): 9.6 (2.8)^		^Mean (SD): 5.6 (2.4)^	
Variable	VIMP	Variable	VIMP	Variable	VIMP
Income	10.681	Income	4.442	Income	11.549
Work hours/week	1.248	BMI	2.667	Work hours/week	0.703
Height	1.011	IL-2sr	2.039	Carotid flow velocity	0.678
Total MET/week	0.743	Waist circumference	1.926	Moderate MET/week	0.521
Carotid distensibility coefficient	0.715	Height	1.269	Carotid distensibility coefficient	0.519
Physical activity hours/week	0.648	Hip circumference	1.219	BMI	0.488
Alcohol use	0.584	Plasmin-antiplasmin complex	0.533	Height	0.481
Transportation MET/week	0.433	TNFsr	0.53	Total MET/week	0.475
Serum glucose	0.414	Work hours/week	0.509	Alcohol use	0.447
Moderate MET/week	0.408	Highest weight in 3 years	0.503	GFR	0.436

TNFsr: Tumor necrosis factor-alpha soluble receptor; MET: Metabolic equivalent of task; IL-2sr: Interleukin 2 soluble receptor; BMI: Body mass index; GFR: Glomerular filtration rate; VIMP: Variable importance; SD: Standard deviation

*z-scores adjusted age, gender, education and race were used

The performance indices for the RSF and RF models on the testing (validation) dataset are presented in Table 4. The Harrell’s C-index of RSF model for incident dementia was 0.81 while the Brier score was 0.04. RMSE and MAE for RF models for measures of cognitive function were less than 0.65 and 0.50, respectively, while R-squared was between 0.34 to 0.41.

**Table 4:** The performance indices of RSF and RF models for incident dementia and measures of cognitive function, respectively.

Random Survival Forest Model		Harell’s C-index	0.81
		Brier score	0.04
Random Forest Model	CASI score	R-squared	0.41
		RMSE	0.64
		MAE	0.48
	Forward digit span	R-squared	0.34
		RMSE	0.67
		MAE	0.46
	Backward digit span	R-squared	0.39
		RMSE	0.65
		MAE	0.50

CASI: Cognitive Abilities Screening Instrument; RMSE: Root mean square error; MAE: Mean absolute error

## Discussion

Using a high dimensional data-driven deep phenotyping approach, this study identified a specific set of important cardiovascular features that correlate with incident dementia and measures of cognitive dysfunction in a well characterized multi-ethnic cohort. Many of the phenotypic correlates of dementia in this study have been also linked to cardiovascular pathologies and include age, markers of atherosclerosis, physical activity, metabolic syndrome (coronary artery calcification, liver to spleen attenuation ratio, carotid IMT, pulse pressure and measures of physical activity), subclinical myocyte damage (hsTroponin), myocardial stress (hsTroponin, NT-proBNP), inflammation (TNFsr and IL-2sr) and vascular function (aortic strain). History of cancer as a major comorbidity associated with inflammation was also among the strongest correlates of dementia. There was significant overlap between top correlates of dementia among older participants and the entire cohort. Genetic predisposition (apolipoprotein E) appeared among top correlates of dementia in older adults but not for the entire cohort. Moreover, socioeconomic status, level of physical activity and body size, vascular function, inflammation, alcohol use, glucose metabolism and kidney function were associated with measures of cognitive function. Another important finding is the high degree of correlation between subclinical cardiovascular disease, demographics and traditional cardiovascular risk factors with dementia and cognitive function as shown by the relatively high C-index and R-squared values for the RF models.

The mixed form dementia is the most common form of dementia, and in most cases, it is clinically impossible to distinguish between vascular and non-vascular etiologic components of dementia [1, 3, 5]. In fact, VCID represents a broad spectrum of cognitive alterations ranging from mild cognitive impairment to vascular dementia, encompassing pathologies from failing to cope with biological insults because of vascular disease, Alzheimer biology, metabolic dysregulation and immune insults, ultimately leading to cognitive decline [1]. Earlier studies emphasized the role of multiple small and large infarcts in the pathogenesis of dementia [25] but more recent data highlight the importance of alterations in small blood vessels as the epicenter of VCID, and suggest that subtle but persistent vascular injury lead to progressive cognitive dysfunction [26–28]. Cerebral hypoperfusion in the setting of carotid artery stenosis or heart failure has also been linked to cognitive impairment [29, 30]. In this regard, we have also reported the association of NT-proBNP and left ventricular hypertrophy with dementia [11, 12] in the same population as this study. Furthermore, in previous pathological studies, cortical microinfarcts (less than 1mm, not visible to the naked eye) secondary to small vessel disease have been associated with dementia [3, 31]. These microinfarcts are also frequently seen in patients with Alzheimer disease [3, 32]. Imaging based studies have also shown that enlarged perivascular spaces as the result of inflammation, hypertension, or changes in perivascular flow are associated with cognitive decline [3, 9, 10, 33, 34]. These pathogenetic mechanisms associated with vascular diseases, cardiac pathology and inflammation may explain our findings regarding the top correlates of dementia in this study. Another notable finding of our study is the association of hepatic steatosis with incident dementia. We have previously reported the association of liver fibrosis assessed by magnetic resonance imaging with cardiovascular events [35]. However, this is the first study to show the link between subclinical liver steatosis and incident dementia. The important association of physical inactivity with incident dementia is in line with previous studies in the literature [36]. Similar to our study, others have also shown that patients with a history of cancer are at higher risk for developing dementia [37]. The high C-index (0.81) evidenced in our study also highlights the posited tight association between pathways leading to dementia and cardiovascular diseases.

In this study, income level was the strongest correlate of cognitive dysfunction. This is in line with previous studies reporting the link between lower socioeconomic status and dementia of any cause [38, 39]. Interestingly, many of the top correlates of cognitive decline in this analysis were modifiable risk factors such as physical activity and body composition while the top correlates of incident dementia were subclinical cardiovascular phenotypes such as CAC score, hsTroponin or carotid IMT, that are considered distal to disease initiation and closer to clinical dementia onset. This may have implications for prevention of VCID and dementia by early control of modifiable risk factors. The potential implication of our findings is further supported with increasing evidence suggesting that some patients with mild VCID have the potential to regain normal cognition [40–42].

In this study, we used a deep phenotyping approach to identify the top correlates of dementia and cognitive dysfunction among hundreds of variables collected as part of a large prospective cardiovascular study. Large epidemiological cohorts offer an invaluable opportunity to explore the pathogenesis, prevention and treatment of clinical conditions such as dementia and cognitive decline; however, such large amounts of data accumulated over time come with unique analytic challenges because conventional statistical methods such as Cox proportional hazard regression modeling, are limited for data mining purposes due to intercorrelation and nonlinearity among variables, and the possibility of overfitting [13]. Furthermore, conventional methods utilize hypothesis driven approaches that while essential to establishing causation chains, have limited ability in the discovery of novel biomarkers. Methods such as RSF and RF are powerful tools to handle the challenge created by very large number of variables and are complementary to established methods based on *a priori* hypotheses [14, 43].

Contemporary literature presents a limited number of studies employing machine learning approaches to investigate dementia, mostly focusing on algorithmic performance in dementia prediction rather than on the elucidation of its risk factors [44]. Notably, studies targeting risk factor identification predominantly rely on brain imaging data and cognitive assessments derived from questionnaires and neuropsychological tests as potential dementia correlates [45, 46]. Ding et al. developed a machine learning model within the Framingham Heart Study, utilizing clinical risk factors, neuropsychological tests, and brain magnetic resonance imaging (MRI) metrics to forecast dementia onset, which ranked age as the preeminent dementia predictor, succeeded by MRI and neuropsychological indices [45]. Moreover, blood pressure emerged as the sole additional clinical marker linked to dementia incidence; nevertheless, this study was constrained by a limited spectrum of clinical risk factors and the exclusion of blood biomarkers and alternative imaging modalities. The prognostic aspect of dementia has also been explored through machine learning, as exemplified by Mostafaei et al., who implemented trio machine learning algorithms to pinpoint mortality predictors among dementia patients within the Swedish Registry for Cognitive/Dementia Disorders [47]. Analogous to age’s predictive primacy for mortality, various cardiovascular risk factors, including body mass index, heart failure, atrial fibrillation, hyperlipidemia, hypertension, diabetes, cancer, liver disease, and atherosclerosis, were found to be associated with mortality in dementia patients, paralleling our identification of similar correlates for incident dementia or cognitive function.

### Limitations

The main limitation of our study was the ascertainment of dementia from hospital discharge diagnoses using ICD codes which potentially underestimates the incidence of dementia, given that outpatient diagnoses can be missed. However, this method has been validated in MESA for diagnoses of clinically established dementia and has been used in several previous studies [11, 12, 18]. Additionally, the present study lacked information concerning the subtypes of dementia prevalent in the MESA cohort, such as Alzheimer’s dementia, vascular dementia, Lewy body dementia, among others. Consequently, an examination of the distinct associations associated with each subtype of dementia was not feasible [18]. Moreover, the absence of an additional external dataset, designated as a replication cohort, further constrained our ability to validate the observed outcomes. As a result, it is anticipated that forthcoming investigations will compare and contrast our findings with those derived from diverse large-scale cohorts.

## Conclusion

Deep phenotyping methods are well suited for discovery of correlates of incident dementia without priori hypotheses in large multi-ethnic cohorts. In our study, markers of myocardial injury and stress, atherosclerosis and inflammation, physical activity, vascular function, and body composition were associated with dementia in our study. Modifiable risk factors of chronic diseases were prominently associated with measures of subclinical cognitive dysfunction.

## Supporting information

S1 Appendix (DOC)
