# Supplementary material for: Deep phenotyping of dementia in a multi-ethnic cardiovascular cohort: The Multi-Ethnic Study of Atherosclerosis (MESA)
Source: PLoS One. 2024 Apr 18;19(4):e0298952. doi: 10.1371/journal.pone.0298952 (PMC11025925; doi:10.1371/journal.pone.0298952)
Supplement: S1 Appendix — (DOC) [file pone.0298952.s001.doc]

**S figure 1**


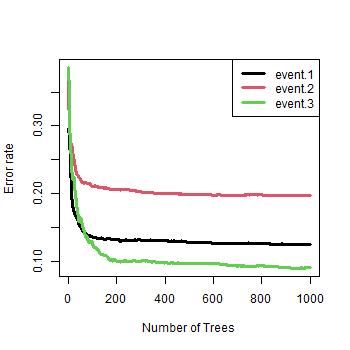


**A**

**B**


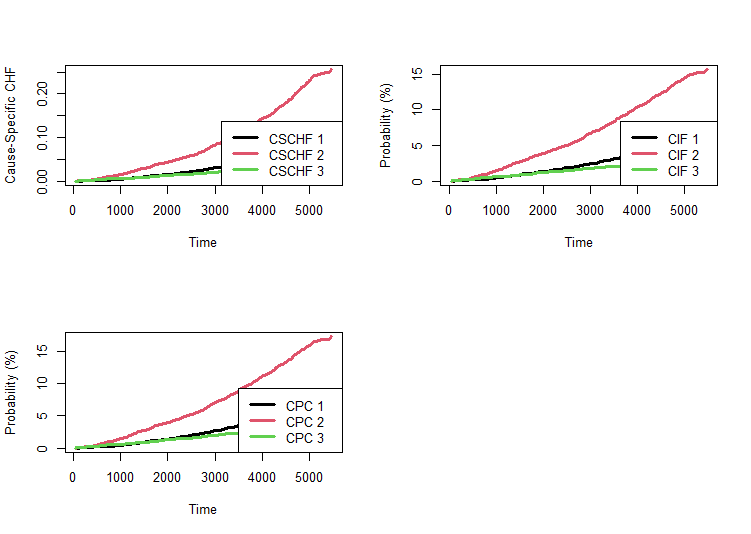


**S figure 2**


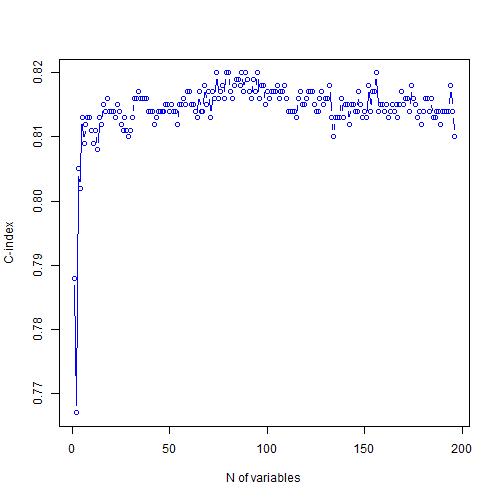


| **S table 1. Sensitivity analysis for top correlates of incident dementia using ntrees=5000 in Random Survival Forest analysis** | |
| --- | --- |
| **Variable** | **VIMP** |
| Age | 0.278 |
| Liver to spleen attenuation ratio | 0.052 |
| hsTroponin | 0.027 |
| Smoking | 0.026 |
| Dorsalis pedis blood pressure | 0.012 |
| CAC volume | 0.011 |
| Right atrial strain | 0.01 |
| Left atrial active ejection fraction | 0.008 |
| Pulse pressure | 0.006 |
| TNFsr | 0.003 |
| VIMP Variable importance; hsTroponin: High sensitivity troponin; CAC: Coronary artery calcification; TNFsr: Tumor necrosis factor-alpha soluble receptor | |

| **S table 2. Top correlates of dementia in participants younger than 62 years old.** | |
| --- | --- |
| **Variable** | **VIMP** |
| Liver to spleen attenuation ratio | 0.211 |
| Perceived discrimination | 0.209 |
| Heart rate variability on ECG | 0.188 |
| Use of Sulfonylureas (diabetes) | 0.165 |
| Dorsalis pedis blood pressure | 0.144 |
| Social support index | 0.139 |
| Physical activity hours per day | 0.123 |
| Left atrial active ejection fraction | 0.122 |
| Diastolic blood pressure | 0.114 |
| Use of angiotensinogen converting enzyme inhibitors | 0.111 |
| VIMP: Variable importance; ECG: Electrocardiogram | |
